# A critical review of population health literacy assessment

**DOI:** 10.1186/s12889-015-1551-6

**Published:** 2015-03-04

**Authors:** Diana Guzys, Amanda Kenny, Virginia Dickson-Swift, Guinever Threlkeld

**Affiliations:** La Trobe Rural Health School, La Trobe University, PO Box 199, Bendigo, 3552 Australia; La Trobe Rural Health School, La Trobe University, P.O. Box 821, Wodonga, Victoria 3689 Australia

**Keywords:** Critical health literacy, Community health literacy assessment

## Abstract

**Background:**

Defining health literacy from a public health perspective places greater emphasis on the knowledge and skills required to prevent disease and for promoting health in everyday life. Addressing health literacy at the community level provides great potential for improving health knowledge, skills and behaviours resulting in better health outcomes. Yet there is a notable absence of discussion in the literature of what a health literate population looks like, or how this is best assessed.

**Discussion:**

The emphasis in assessing health literacy has predominantly focused on the functional health literacy of individuals in clinical settings. This review examines currently available health literacy assessment tools to identify how well suited they are in addressing health literacy beyond clinical care settings and beyond the individual. Although public health literature appears to place greater emphasis on conceptualizing critical health literacy, the focus continues to remain on assessing individuals, rather than on health literacy within the context of families, communities and population groups. When a population approach is adopted, an aggregate of individual health literacy assessment is generally used. Aggregation of individual health literacy fails to capture the dynamic and often synergistic relationships within communities, and fails to reflect societal influences on health knowledge, beliefs and behaviours.

We hypothesise that a different assessment framework is required to adequately address the complexities of community health literacy. We assert that a public health approach, founded on health promotion theories provides a useful scaffold to assess the critical health literacy of population groups. It is proposed that inclusion of community members in the research process is a necessary requirement to coproduce such an appropriate assessment framework.

**Summary:**

We contend that health literacy assessment and potential interventions need to shift to promoting the knowledge and skills essential for critical health literacy at a societal level. The challenge for researchers is to negotiate the myriad of complexities associated with each concept and component required for this task.

In this critical review we explore how the development of health literacy assessment tools reflects the evolution of health literacy definitions. Improving the health literacy of individuals, organisations and communities is identified in health care reform globally as a goal associated with improved health outcomes and health service efficiency [1]. Health literacy interventions at a population level provide great potential for improving health knowledge, skills and behaviours and consequently better health outcomes of communities. However, health literacy assessment predominantly focuses on the literacy and communication skills of individuals in clinical settings. This review examines commonly used assessment tools to determine their appropriateness for assessing the critical health literacy of population groups. We demonstrate the need to develop a framework appropriate for assessing the critical health literacy of population groups, and conclude that health promotion theories and principles provide a useful scaffold for this. We acknowledge that managing the complexities of the multiple concepts in achieving this, is somewhat daunting.

## Background

Early conceptions of health literacy emphasized an individual’s ability to understand and comply with health instructions. This resulted in a focus on functional health literacy, premised on an individual having the requisite reading and numeracy skills, and interactive health literacy, relating to personal communication skills required to engage effectively with health care professionals [2]. Medical health literacy is another term which has been used to describe this, as the fundamental focus is on improving the quality of clinical interactions [3]. Nutbeam [2] further described critical health literacy, which progresses from functional and interactive health literacy. He argued for a wider range and more advanced levels of knowledge and skills that support greater autonomy and personal empowerment in health related decision-making and management. Across the population critical health literacy enhances capacity to act politically to address social and economic determinants of health [2]. It has been noted, however, that this second component of Nutbeam’s description of critical health literacy, the capacity to act politically to address social and economic determinants of health, is a common omission in the literature [4,5].

Critical health literacy facilitates improved individual resilience to social and economic adversity, and contributes to community empowerment [4,5]. Zarcadoolas, Pleasant and Greer [6] propose a health literacy model which incorporates functional literacy (reading, writing, speaking and numeracy), science literacy, civic literacy and cultural literacy. This model expands upon Nutbeam’s conception of critical health literacy, providing further detail of its multiple facets. Scientific literacy relates to an understanding of science and technology, as well as acknowledgment that these can change rapidly. Civic literacy incorporates knowledge of social processes, social capital, social cohesion and media literacy skills. Whereas cultural literacy refers to recognition of collective beliefs and practices, and understanding how social identity influences behaviours and decision making [6]. This expanded model addresses some of the concerns expressed by Tones [7], about the necessity for the evolving conception of health literacy.

The purpose of a critical review is to present, analyse and synthesise material from a range of appropriate sources, to produce a hypothesis or a model, rather than produce an answer [8]. This method is consistent with interpretivist philosophy and constructivist orientation, as knowledge is produced through the process of considering the views of others. Compared to other forms of literature reviews, a critical review is less formally structured, and is usually narrative or chronological. It does not provide a survey of all of the available literature on a topic; rather it demonstrates extensive research with the objective of identifying significant contributions to conceptual development in a field, for critical evaluation [8]. It is only in the last year several systematic reviews of health literacy assessment tools have been published recently [9-11], along with a paper discussing the evolution of health literacy tools [12]. We therefore encourage those seeking a more detailed review the health literacy assessment tools available, to read these.

## Discussion

Inconsistent conceptualisation and definition of health literacy is frequently discussed in academic literature. It has been suggested that this results from concepts having a cluster of attributes, rather than a strict set of attributes, that are subject to change as they are prioritised differently by different groups of people [5]. The most frequently cited definitions of health literacy are sourced from the American Medical Association, the Institute of Medicine and the World Health Organisation (WHO), which focus on the skills necessary for an individual to obtain, process and understand health information and services facilitating healthy decision making [4]. However, the WHO Commission on Social Determinants of Health [13] health equity report called for expansion in the scope of health literacy to include the ability to understand and communicate information related to the social determinants of health. Begoray and Kwan [14] developed an operational definition of health literacy by reviewing the shared elements of existing definitions to identify four broad health literacy skills: the degree to which people are able to access; understand; evaluate; and communicate information in order to promote and maintain health across the life-course, across a range of contexts.

A number of reviews have been undertaken in an effort to clarify the definition and conceptualization of critical health literacy in the belief that this will enable greater progress toward its achievement. Public health literature, rather than clinical literature, appears to place greater emphasis on conceptualizing critical health literacy. Yet even within public health literature, the focus of health literacy continues to primarily assess individuals, rather than consider health literacy within the context of families, communities and population groups. Sykes et al. [5] characterised critical health literacy as advanced personal skills including health knowledge, effective interaction between service providers and users, informed decision making and empowerment, including political action. An understanding the social determinants of health, critical appraisal of information and collective action, were identified by Chinn [15]. However, Sykes et al. [5] report that health professionals and policy makers believe that it is the commitment from health practitioners to provide accessible information and to engage in shared decision making, which is required for the emergence of critical health literacy.

Over the last five years reference to action at a social and political level, and population level empowerment associated with conceptualisation of critical health literacy, have declined [5]. This conceptualisation of critical health literacy focuses on the relationship between services and individuals. The emphasis becomes an individual’s ability to navigate services, communicate effectively and confidently with a health professional, including constructively questioning or challenging them when necessary [5]. The Health Literacy Pathway Model developed by Edwards, Wood, Davies and Edwards [16] exemplifies this conception. This model describes the trajectory of an individual’s development of health literacy, as they seek, engage and act on health information in relation to their health condition [16]. Although useful in improving the health outcomes of individuals, adopting only this view of critical health literacy must be challenged, as it perpetuates an emphasis of responding to ill health, rather than acknowledging the social determinants of health and taking preventative action through promoting health.

Assessment of health literacy is recognized as an important consideration in delivering appropriately tailored effective health care and achieving better health outcomes. Increased health knowledge is thought to positively influence health behaviours and consequently this is reflected in health status. However, health literacy assessment tools continue to primarily focus on individuals and are slow in shifting shift from a medical perspective towards a societal one. It has been argued for a distinction to be made between public and individual health literacy [17].

The need to develop new tools to assess health literacy more broadly has been recognized. More recent tools have sought to address other dimensions that impact on health and health literacy [18-20]. Yet a greater focus on heath literacy outside of healthcare settings is required, as this is where there is the greatest potential to impact on health behaviours to prevent or reduce ill health [4]. We contend that a distinction between how public or community health literacy and personal health literacy is assessed is necessary. We critically examine currently available health literacy assessment tools to identify how well suited they are in addressing health literacy beyond the individual and beyond clinical care settings.

The conceptualization of health literacy assessment tools is understandably consistent with the conceptualization of health literacy itself. A number of tools have been developed to assist in assessing health literacy in health care settings, which can be broadly categorized as assessing word recognition, reading comprehension and functional health literacy through informal measures. Informal methods may include observation of behavior such as forgetting eyeglasses necessary for reading information during visits; submitting incomplete forms; missing appointments, diagnostic tests or procedures; or the incorrect administration of medication or treatment advice [21].

The most commonly used tools reported in the literature are the Rapid Estimate of Adult Literacy in Medicine-Short Form (REALM-SF), which tests reading ability through word recognition and pronunciation [22]; The Test of Functional Health Literacy in Adults (TOFHLA), which requires patients to read and complete missing sections of selected passages of information to measure reading comprehension, as well as to read and apply the information on prescription labels and appointment slips to assess numeracy [23]; and The Newest Vital Sign (NVS) a quick assessment of reading comprehension and numeracy, requiring patients read an ice cream label nutritional label, then answer 6 problem-solving questions [18,21].

Discussion of the use of REALM and its short form version REALM-SF to assess health literacy in primary care settings has appeared in literature since the early 1990s. Prior to the introduction of REALM, the Wide Range Achievement Test-Revised (WRAT-R) was used to assess functional literacy skills, described as the ability to read, write and make calculations to deal with everyday situations [22,23]. Moving beyond testing word recognition and pronunciation of health related words, TOFHLA was developed in the mid 1990s, assessing reading comprehension using documents and materials found in health care settings [23]. Another health literacy assessment tool which focuses on assessing reading comprehension and numeracy skills, is the NVS, which was developed almost 10 years after TOFHLA. The key advantage claimed through the use of the NVS over TOFHLA is that it takes less time to administer [24]. Time to administer these health literacy assessments, as well as the potential to embarrass patients through the use of such assessments, lead to the development of using screening questions for the quick and unobtrusive identification of those with limited or marginal functional health literacy skills [25-27]. The use of a single question, asking the patient how confident they were in filling out medical forms, was advocated [26,27]. However, all of these health literacy assessment tools clearly focus on functional literacy assessment of individuals for clinical care, providing little assistance for assessing critical health literacy or in a population context.

Some population approaches have attempted to move beyond assessing individuals, yet the emphasis remains on functional health literacy and a medical view of health, rather than the broader social view of health and wellness. The Demographic Assessment for Health Literacy (DAHL) is used to impute limited health literacy and estimate the association with indicators of health status [28]. Another population approach identified uses a similar model based on social demographics to predict inadequate functional health literacy [29].

More recently developed assessment tools have endeavoured to adopt a broader conceptualisation of health literacy. The recognition that health outcomes of patients with diabetes were inconsistent with their functional literacy level highlighted that a person’s ability to read health information was not singularly necessary for optimal self management [30]. Ishikawa et al. [30] developed a tool they believe measures functional, communicative and critical health literacy by assessing a person’s ability to obtain, critically analyse, and use health information to positively participate in their own health care. This was presented as a self reported, four point scale questionnaire, which they felt was easy to deliver in a clinical setting. Their findings suggests that each type of health literacy may impact on health outcomes in different ways, as the skills necessary to read the information may be less important in managing diabetes than the skills of extracting, communicating, and applying information.

Another recent development in assessing health literacy is the Health Literacy Questionnaire (HLQ). The HLQ is reported as having multiple functions, including identifying the needs and capabilities of individuals, describing the health literacy of populations, and evaluating outcomes of public health and clinical interventions designed to improve health literacy [18]. The HLQ assesses nine indicators of health literacy which reflect the experience of people attempting to access, understand and using health information as well as engage with health services, reflecting perspectives sought from the general population, health care providers and policymakers. Individuals are asked to self rate their active self management of their health; social support for health; access to sufficient information to manage their health; ability to find good health information; ability to judge the quality of health information; ability to understand health information well enough to act on this; ability to navigate the healthcare system; ability to actively engage with healthcare providers; and the degree to which they feel understood and supported by healthcare providers [18].

The All Aspects of Health Literacy Scale (AAHLS) is another recently developed tool, which aims to measure functional, communicative and critical health literacy. The AAHLS is reported to identify an individual’s health literacy support needs, highlight the strengths and capabilities an individual has, provide population level information and may be used to evaluate the impact of local patient education initiatives [31]. The AAHLS is a self-reported scaled questionnaire designed to assess an individual’s ability to read health information; to write; to gather, process and appraise information; access support networks and interact successfully with health providers. The scale includes questions to assess an individual’s willingness and ability to assert individual autonomy in relation to healthcare decisions, as well as if they hold a positive belief about the contribution to the health outcomes of the broader community made by individuals.

The European Health Literacy Survey Questionnaire (HLS-EU-Q) was developed to measure and compare health literacy in populations in selected countries in Europe. The health literacy assessment tool developed by the HLS-EU Consortium is presented as being different from other tools as it is grounded in public health, and addresses the key processes of accessing, understanding, appraising and applying health related information within healthcare, disease prevention and health promotion [32]. Sorensen and colleagues argue that the key limitations of existing tools result from focusing on single or selected components of health literacy, rather than a comprehensive conceptualization of health literacy; as well as on personal attributes, rather than those of the population. The HLS-EU-Q differs from most other health literacy assessment tools, as the stated aim was to measure the health literacy of general populations, rather than specific patient groups [32]. The tool measures an individual’s response to a questionnaire consisting of 47 health literacy related items. Although a continued emphasis on healthcare and disease prevention and less on health promotion is an acknowledged limitation in the design of the tool [32].

Other attempts that focus on measuring health literacy from a public health, rather than medical perspective, are appearing in recently published literature. One study described the use of a short survey tool, which addressed the different domains of health literacy, focusing on adolescents in their daily life context of family and peers [33]. A self reported response was required to eight items that represented functional, interactive and critical health literacy. Two items relating to each of the three domains of health literacy were included, as well as two additional items relating to functional health literacy; differentiating between understanding and finding health-relevant information [33].

These developments demonstrate a response to the ongoing evolution in assessing health literacy, yet the focus remains on the health literacy of individuals or the collation of individual data. The progression of development in the health literacy tools described demonstrates acknowledgement of the complexity of health literacy as a concept, as well as the necessity to incorporate this complexity within assessment. Moving beyond individual functional health literacy competencies in medical settings, to understand the intricacies of critical health literacy in the context of the everyday life of individuals, families and communities provides the impetus to affect great benefit in the health of individuals and populations [34]. Key deficits in current measures of health literacy as summarized by Pleasant et al. [34] include a focus on a single or narrowly defined dimension of health literacy, rather than integrating multiple dimensions; failure to consider health literacy as a public health issue; and a lack in testing or advancing an underpinning theory of health literacy. Addressing these deficits and other attributes required of proposed health literacy assessment mechanisms include the use of measures that are appropriate to the context in which they are being used, reflect more recent definitions and understanding of health literacy and prioritise social research and public health applications [34]. This is particularly relevant in relation to assessing the most challenging and frequently overlooked aspects of health literacy, being critical health literacy and the health literacy of communities or population groups.

Fundamentally public health is concerned with protecting and promoting the health of populations rather than the provision of individual care. Therefore defining health literacy from a public health perspective places greater emphasis on the knowledge and skills required to prevent disease and for promoting health in everyday life [33,35]. A public health approach recognises health literacy as an asset which can be built [34] through action at an individual, service and societal level [1,2]. Building health literacy requires more than citizens acquiring basic health knowledge. Discourse focused on health literacy from a public health perspective frequently refers to improvement in health literacy of ‘individuals and communities’ or ‘populations’ [3,34], yet there is an absence of discussion in the literature of what a health literate population looks like.

Health literacy is acknowledged as a complex multifactor concept. Assessing the impact of numerous complex variables that contribute to the development and use of this knowledge and skills is daunting. Given the complexities that influence daily life decisions which impact on health, a systematic assessment of health literacy is necessary [3]. The challenge is to move beyond a focus of functional and interactive health literacy, to focus on critical health literacy, as well as moving beyond the collation of individual assessments to describe the health literacy of groups and populations.

Health promotion provides a useful scaffold to assess the critical health literacy of population groups. McQueen and Kickbusch [36] describe health promotion as the avant- garde of public health, shifting the focus of public health away from disease to focus on the modern world challenges of creating and maintaining healthy populations. The elemental principle of empowering individuals and communities to achieve optimal health outcomes is embedded within both health literacy and health promotion, intrinsically enmeshing these concepts, leading to the call for health literacy to be more explicitly linked to health promotion theories and models [4]. Empowerment, that is the redistribution of unequal power, is central in addressing health determinants, and consequently health outcomes [37]. Perceiving health literacy as a determinant of health facilitates appropriate and customised interventions that successfully advance the public’s health [19]. Such interventions constitute health promotion activity.

Assessing the health literacy of communities or populations cannot simply or conveniently be addressed through nominating health promotion theory as guiding actions or activity. Researchers need to identify and clearly explain the framework on which health literacy assessment tools are based [14]. Adding to the complexity of this challenge is the number of theoretical foundations which contribute to health promotion, rather than one accepted fundamental grand theory [36]. Perhaps acknowledging the layers of complexity relating to health promotion theories, a public health approach, the domains of health literacy and a population perspective, will enable them to be neatly packed up like Russian nesting dolls, to facilitate the development of a suitable framework to assess the critical health literacy of communities.

The tools developed thus far to assess health literacy reflect another potential limitation, as their development has predominantly been shaped by education, communication and health care experts, with little but cosmetic input from health consumers. Genuine, collaboration with members of the general public, who have a range of literacy and health literacy levels, is required to develop an appropriate framework to assess the critical health literacy of communities. People who directly experience the barriers and benefits of health literacy are the true experts in health literacy, therefore involving the public in the research process is essential [19]. How this is best achieved in a genuine, non tokenistic manner adds a further layer of complexity to the Russian nesting doll analogy. The development of a framework to assess the health literacy of communities needs to integrate the views of community members, a focus on the critical domain of health literacy, health promotion theories, and a public health approach. The creative integration of these components should result in the coproduction of responsive, community informed, health literacy measures.

## Summary

The emphasis of health literacy assessment has focused on the functional health literacy of individuals in clinical settings. Yet, addressing health literacy in the community provides great potential for improving health knowledge, skills and behaviours resulting in better health outcomes. We have critically reviewed a number of health literacy assessment tools available internationally, and demonstrated how that they are unsuited for assessing critical health literacy at the societal level. We contend that the focus of health literacy assessment and potential intervention needs to shift to promoting the knowledge and skills essential for critical health literacy at a societal level. The need to develop a framework appropriate for assessing the critical health literacy of population groups is patently apparent. We concur with others who have an interest in this area that health promotion principles and theories provide a useful scaffold for developing the required assessment framework, particularly as possible interventions which result from such an assessment are health promotion activities. We further propose that inclusion of community members in the research process is necessary to coproduce such an assessment framework. The challenge for researchers is to negotiate the myriad of complexities associated with each concept and component of this task.
